# Surgical Outcomes of Inferior Oblique Myectomy in Unilateral Congenital Superior Oblique Palsy with or without Trochlear Nerve

**DOI:** 10.1371/journal.pone.0156872

**Published:** 2016-07-08

**Authors:** Ji Eun Lee, Hee Kyung Yang, Jeong-Min Hwang

**Affiliations:** Department of Ophthalmology, Seoul National University College of Medicine, Seoul National University Bundang Hospital, Seongnam, South Korea; Sun Yat-sen University, CHINA

## Abstract

**Objectives:**

To compare the surgical outcomes of inferior oblique (IO) myectomy in congenital superior oblique palsy (SOP) according to the presence of the trochlear nerve identified with high-resolution MRI.

**Data Extraction:**

Forty-one congenital SOP patients without a trochlear nerve (absent group) and 23 patients with a trochlear nerve (present group) who underwent IO myectomy as the primary surgical treatment were retrospectively reviewed. “Motor success” was defined as postoperative ipsilateral hypertropia ≤ 4 prism diopter (PD). “Head tilt improvement” was regarded as postoperative angle of head tilt < 5 degrees (°). Success rates for motor alignment and head tilt improvement, cumulative probabilities of success, and factors influencing surgical responses were evaluated.

**Results:**

The cumulative probabilities of motor success at 2 years after IO myectomy were 92% in patients with a trochlear nerve and 86% in patients without a trochlear nerve (*P* = 0.138). The cumulative probabilities of undercorrection and recurrence of hypertropia after 2 years were 0% in the present group versus 21% in the absent group (*P* = 0.014). The cumulative probabilities of persistent head tilt after 2 years were 14% in the present group and 20% in the absent group (*P* = 0.486). A younger age at operation was associated with reduced probabilities of motor success and head tilt improvement (*P* = 0.009, *P* = 0.022 respectively). A greater preoperative angle of head tilt was associated with persistent head tilt after surgery (*P* = 0.038).

**Conclusions:**

Congenital SOP without a trochlear nerve had a higher risk of hypertropia undercorrection after IO myectomy compared to patients with a trochlear nerve. A younger age at operation and larger preoperative head tilt was related to poor outcomes.

## Introduction

Recent advances in high-resolution thin-section magnetic resonance imaging (MRI) techniques now provide another means of evaluating the pathogenic mechanism of congenital superior oblique palsy (SOP) allowing direct identification of congenital trochlear nerve agenesis in patients with superior oblique (SO) muscle hypoplasia. [1–3] Our recent studies introduced novel aspects in the classification of congenital SOP according to the presence of the trochlear nerve and demonstrated clinical differences between these groups in details. [2] In patients with congenital SOP, 73% showed ipsilateral trochlear nerve absence and a variable degree of SO hypoplasia, while the remaining 27% had a normal morphologic SO volume and the trochlear nerve on both sides, suggesting distinguished etiologies. [2] However, regardless of the etiology, the surgical treatment for congenital SOP is based on ocular motility patterns including ductions and versions, muscle restriction or torsional changes. [4, 5]

Surgical management of SOP consists of strengthening the SO muscle, weakening the inferior oblique (IO) muscle of the paretic eye, or operating on the vertical rectus muscles, as well as a combination of these operations. [4–7] IO weakening procedures are frequently applied as surgical options if secondary IO overaction is prominent without the evidence of SO tendon laxity or significant SO weakness. [4–6, 8–11] There have been numerous reports about the effects of IO myectomy in reducing the vertical deviation as well as eliminating the anomalous head postures. [5, 9, 11, 12] Toosi and von Noorden documented that IO myectomy was a simple, safe and effective surgery for SOP accompanying IO overaction. [9] Helveston and associates also described that IO myectomy had low complications such as an iatrogenic Brown syndrome and great efficacies to treat the symptoms from SOP as an initial procedure. [5] In addition, Shipman and Burke reported that IO myectomy resulted in the greatest reduction of hypertropia in the primary position up to 15 prism diopters (PD) or more with better predictable long term outcome compared to IO recession. [10] Ghazawy and associates identified that IO myectomy improved SO underaction irrespective of the accordance of primary or secondary IO overaction. [12] Hence, uncombined IO myectomy has been commonly preferred as the initial procedure, especially in SOP patients with overaction of the ipsilateral IO and preoperative hyperdeviation of 10 to 15 prism diopter (PD) or more. [9–11]

There have been several studies about surgical results between various IO weakening surgeries in SOP. [10, 11, 13–15] In contrast, the comparable long-term outcomes of IO weakening surgery for the treatment of congenital SOP according to the 2 distinct pathologic mechanisms; the presence or absence of the trochlear nerve, have not yet been documented in the literature. It would be practical to be able to predict surgical outcomes according to pathologic causes, such as SO atrophy and absence of the trochlear nerve. Herein, the present investigation was conducted to compare the surgical outcomes of IO myectomy as the primary intervention in congenital SOP, depending on the presence of the trochlear nerve identified with thin-section high-resolution MRI. In addition, factors influencing the surgical outcomes were also evaluated.

## Materials and Methods

### Patient characteristics

A retrospective review of medical records was performed on consecutive patients who underwent their first operation under the care of one surgeon (J-MH) for unilateral SOP with high-resolution thin-section MRI images at Seoul National University Bundang Hospital between January 2012 and May 2015. Study subjects were included if they had the confirmatory evidences of unilateral congenital SOP including: presence of underdepression and/or overelevation in adduction on the affected side, positive Parks-Bielschowsky three-step test results, large fusional amplitudes of vertical deviation, a reliable history or photographic records of long-standing strabismus or anomalous head posture from infancy or early childhood, and the absence of a reversal of the hypertropia in any of the nine diagnostic gazes or head tilt, or a combination thereof. Patients who underwent IO myectomy as the first surgical treatment were included. The surgical indication for IO myectomy in patients with unilateral congenital SOP was 1) constant hypertropia in the primary position of ≤ 20 PD and no sign of superior rectus (SR) contracture which was confirmed by the result of an intraoperative forced duction test, and/or 2) contralateral head tilt ≥ 5 degrees (°) with the existence of prominent facial asymmetry as a result of prolonged head tilt. Even when the maximum amount of hypertropia was over 20 PD, IO myectomy was selectively conducted if fusional control was good in the primary position and there was no sign of SR contracture according to the results of the forced duction test. IO myectomy was conducted as follows: A fornix incision was made at the inferotemporal quadrant of the bulbar conjunctiva. After dissecting Tenon’s capsule, the IO muscle was isolated under direct vision. The IO muscle was dissected of its surrounding intermuscular septa from its insertion to near the temporal border of the inferior rectus muscle. Two hemostatic forceps were applied to separate the IO muscle and the IO muscle was excised between the hemostats. [16]

We excluded patients if there was primary overaction of the IO on the affected side, any etiologies related to acquired diseases such as a history of head trauma, strabismus surgery, or other simulating causes including craniofacial anomalies, central nervous system diseases and myogenic torticollis. Patients with bilateral SOP, incomplete medical records and a follow-up period of less than 6 months were also excluded. Approval to conduct this study was obtained from the Institutional Review Board of Seoul National University Bundang Hospital. All clinical investigation was conducted according to the principles expressed in the Declaration of Helsinki. Informed consent was not given, as patient records and information were anonymized and de-identified prior to analysis.

### Identification of the Trochlear Nerve on MRI

MRI was achieved using a 3-Tesla system (InteraAchieva; Philips, Best, The Netherlands) with 8-channel sensitivity encoding head coil, initially with T2-weighted imaging of the entire brain and the orbit and subsequently with high-resolution cranial nerve imaging of the brain stem. General aspects of the MRI protocol have been described elsewhere. [1–3] Initially, the orbital imaging for the status of the extraocular muscles was performed with the turbo spin-echo technique, without the use of defined targets for visual fixation. The imaging plane was set coronal orthogonally to the anterior cranial base and the scanning range was permitted from the most anterior part of the eyeball to the sellar region. In addition, high-resolution cranial nerve imaging to visualize the cisternal segment of the trochlear nerve was scanned with a 3-dimensional balanced turbo field echo sequence at the pontomesencephalic junction, containing the inferior margin of the inferior colliculus, which is known to be the site of the root exit of the trochlear nerve. The scanning plane was optimized to an oblique axial direction perpendicular against the long axis of the aqueduct, which was nearly parallel to the course of the trochlear nerve. The sequence parameters were as follows: repetition time/echo time, 9.9/5.0 milliseconds; flip angle, 60°; field of view, 150X150 mm; matrix, 500X500; section thickness, 0.25 mm; number of slices, 60; sensitivity encoding (SENSE) factor, 2; number of signal averaging, 2; and acquisition time, 7 minutes and 14 seconds. The voxel size was0.3X0.3X0.25 mm. [1] Children younger than 6 years were sedated by chloral hydrate. The extraocular muscles were evaluated visually on the orbital coronal sections to determine morphologic asymmetry, especially for the SO muscle. The presence or absence of both trochlear nerves was determined visually on high-resolution cranial nerve images. Definite identification of the trochlear nerve on MRI was based on the following findings: (1) identification of the root exit zone of the nerve at the inferior margin of the inferior colliculus and (2) presence of a curvilinear, nonbranching structure in the perimesencephalic cistern coursing in the anterolateral direction toward the ipsilateral tentorium. In several aplastic cases, images were reformatted in the various oblique planes to fit the course of the trochlear nerve. [1, 17–19]

### Preoperative Evaluation

Clinical characteristics were recorded as follows: gender; birth history; family history; initial presenting signs or symptoms (chief symptom), classified such as head tilt, ocular deviation, or diplopia; age at onset of signs or symptoms; best-corrected visual acuity; refractive errors; presence of amblyopia, defined as a difference of 2 lines or more between monocular visual acuities; and anisometropia of more than 1.50 diopters.

All study patients underwent preoperative and postoperative ophthalmologic examinations. Examinations included prism and alternate cover measurements in the 6 diagnostic positions of gaze with right and left head tilts including Parks-Bielschowsky three-step test, and the maximum amount of deviation observed preoperatively was recorded. Laterality of the paretic eye, fixation dominance, dissociated vertical deviation (DVD) and combined horizontal strabismus were also noted. Ocular motility assessment for the oblique muscles was graded based on a subjective scale (0–4) of underaction (-) or overaction (+). SR muscle overaction or contracture, or Jampolsky syndrome was defined as a vertical deviation of 12 PD or more in the primary position, equal or larger hypertropia with ipsilateral head tilt than in the primary position, more than 5 PD hypertropia of the affected eye with ipsilateral abduction, hypertropia in all upgazes, and overaction of the contralateral SO muscle of +1 or more. [20, 21]

Quantification of compensatory head tilt posture was obtained using clinical and photographic methods. First, we directly assessed the head tilt posture and degree of head tilt while the patient was fixating on a distant object (clinical measurement). The other method was determined from photographs taken in a sitting position while the patient was fixating on a distant target (photographic measurement). Using Photoshop (Adobe Photoshop CS4, Adobe Systems Inc, CA, USA), a vertical midline was drawn perpendicular to the floor of the digital image and the other was drawn thorough the vertical axis of the face, as the method originally described using the goniometer. [22] The amount of anomalous head posture can be measured directly from the angle between these two lines. An angle of more than 5° was indicated as a presence of head tilt. However, as this study was retrospective, we could not obtain both clinical and photographic measurements from all patients. So, we compared the consistencies of those values from 22 patients who had both measurements preoperatively. Intra-class correlation coefficient (ICC) was 0.742 (95% CI 0.379, 0.893, *P* = 0.002) and the limit of agreement (LoA) was -16.051 to 17.230 degrees. Although the absolute values of LoA were relatively high and the range was wide, the Bland-Altman plot showed that only 2 cases were outside of the range. Hence, the consistency of the 2 methods was acceptable and we used either one of the preoperative maximum angles for analysis. The presence of facial asymmetry was graded subjectively by 2 independent observers on full-face frontal photographs obtained while the patient fixated on a distant target at the primary position. Sensory status was examined in cooperative patients using the Randot stereoacuity test. A fine stereoacuity under 100 seconds of arc (arcsec) with the Randot stereoacuity test was regarded as good stereopsis.

Data collection included the age at surgery, follow-up duration and results of intraoperative Guyton’s exaggerated traction test. [23] Measurements acquired during the preoperative visit nearest to the date of surgery were used for analysis.

### Main Outcome Measures

Postoperative vertical alignment at distance and near in the primary position was measured as well as the angle of head tilt at postoperative months 1, 12, and later. The initial and final results were based on the results measured at the first and latest follow-up visit.

The two main outcome measures were the correction effect on hypertropia in the primary position (motor success) and head tilt (head tilt improvement). First, the criteria for ‘motor success’ was defined as a postoperative ipsilateral hypertropia of ≤ 4 PD. Overcorrection was defined as reversal of hypertropia. Undercorrection was defined as a residual hypertropia of > 4 PD at any time of follow-up. Recurrence was confirmed as a new onset of ipsilateral hypertropia in the primary position after at least 6 months of an initial motor success. If contralateral hyperdeviation of more than 4 PD developed after surgery, which increased with horizontal gaze toward the originally paretic side and was greater on head tilt testing toward the side of postoperative hypertropia, such patients were considered to have a masked bilateral SOP. [24] Second, postoperative head tilt of less than 5° was regarded as ‘head tilt improvement’. The maximum residual angle of head tilt at each follow-up was measured based on the patients’ postoperative photograph. Patients who did not have photographs at each time point were evaluated based on medical records, such as significantly improved or not. Concerning cases with missing data of both methods, we alternatively presented the proportion of improved patients against the number of patients who exhibited preoperative abnormal head posture at each time point. Cumulative probabilities of motor success and head tilt improvement were determined by Kaplan-Meier survival analyses. Factors affecting surgical outcome were also evaluated.

### Statistical Analyses

Statistical analyses were performed with SPSS software version 20 (SPSS Inc, Chicago, IL). Cox analyses were conducted using SAS software version 9.3 (SAS Inc, Cary, NC). Continuous variables were expressed as mean ± standard deviation. The independent *t* test, Pearson’s chi-square tests, Fisher’s exact test, and likelihood ratio test for trend were used to compare the preoperative characteristics between both groups. Pearson’s chi-square tests and Fisher’s exact tests were used to compare the success rates between groups. Kaplan-Meier survival analyses and log-rank tests were used for the comparison of the cumulative probabilities of motor success and persistent head tilt. Cox proportional hazards model with firth’s correction were used to analyze for the predictor variables associated surgical success or adverse outcome. *P* values less than 0.05 were considered statistically significant.

## Results

Of the 215 surgically treated congenital SOP patients, 129 patients who underwent operations other than IO myectomy including vertical muscle surgery with and without IO weakening procedures, Harada-Ito operation, or previous surgery in other medical centers were excluded. Twenty one patients were excluded due to inadequate data collection or loss of follow-up and 1 patient could not determine the presence of the trochlear nerve due to poor image quality of high resolution MRI. Finally, a total of 64 patients were included, with 23 (36%) patients in the present group and 41 (64%) patients in the absent group. All patients of the absent group had an ipsilateral absent trochlear nerve and variable degree of SO muscle hypoplasia, while the fellow eye had an intact trochlear nerve and SO muscle, as previously described. [1] The present group had normal anatomic features of the SO muscle and the trochlear nerve on both sides.

### Patient Characteristics

The preoperative characteristics of both groups are presented in Table 1. Initial clinical signs and symptoms were significantly different between groups (*P* = 0.001), and the most common presenting symptom was ocular deviation in the present group and compensatory head tilt in the absent group. Age at onset of signs or symptoms was younger in the absent group (*P* = 0.031). Age at operation was significantly younger in the absent group (*P* = 0.005). The proportion of patients with good stereopsis (≤ 100 arcsec) was larger in the absent group (*P* = 0.013). The patients with a trochlear nerve had more frequent diplopia (*P* = 0.020), combined horizontal strabismus (*P* = 0.015) and facial asymmetry which tended to be more apparent in the old age group over 5 years old (*P* = 0.054, borderline significance).

**Table 1:** Clinical Characteristics of Patients with Congenital Superior Oblique Palsy Compared between Patients with a Trochlear Nerve (Present Group) versus Those without a Trochlear Nerve (Absent Group).

	Present Group(n = 23)	Absent Group(n = 41)	*P*-value
Age at onset of signs/symptoms (range) (y)	5.8 ± 6.2 (1–25)	2.7 ± 2.9 (1–13)	0.031*
Initial signs/symptoms (chief symptom)			0.001*
Head tilt (%)	6 (26)	26 (63)	
Ocular deviation (%)	9 (39)	13 (32)	
Diplopia (%)	5 (22)	0 (0)	
Incidental (%)	3 (13)	2 (5)	
Onset within 1 year of birth (%)	9 (39)	27 (66)	0.039*
Head tilt within 1 year of birth (%)	13 (57)	27 (66)	0.459
Male gender (%)	14 (61)	28 (68)	0.549
Laterality of SOP			
Right (%)	13 (57)	24 (59)	0.876
Head tilt (%)	19 (83)	38 (92.7)	0.240
Head tilt angle (range) (°)	9.4 ± 8.6 (0–30.2)	14.6 ± 11.8 (0–45)	0.067
Head tilt angle by definition^a^(range) (°)	12.8 ± 8.3 (5–30.2) (16/19)^b^	17.8 ± 10.9 (5–45) (33/38)^b^	0.109
Hypertropia in primary gaze(range) (PD)	17.0 ± 8.4 (0–35)	17.2 ± 6.2 (4–30)	0.914
Facial asymmetry (%)	9 (39)	22 (54)	0.264
Young age (1-4y) (%)	7/12 (58)	18/33 (55)	
Old age (5-25y) (%)	2/11 (18)	4/8 (50)	
*P*-value	0.054	0.819	
Diplopia (%)	5 (22)	1 (2)	0.020*
Refractive error (range) (D)	-0.5 ± 2.9 (-6.3 to 5.2)	-0.2 ± 2.1 (-9.2 to 3.6)	0.638
Anisometropia > 1.50 (D) (%)	2 (9)	4 (10)	>0.999
Amblyopia (%)	4 (17)	5 (12)	0.711
Good stereopsis ≤ 100 (arcsec) (%)	10/20 (50)	15/18 (83)	0.013*
Combined horizontal strabismus (%)	16 (70)	15 (37)	0.015*
Exotropia/esotropia	12 (52) / 4 (17)	14 (34) / 1 (2)	
Paretic eye fixation (%)	3 (13)	6 (15)	>0.999
Dissociated vertical deviation (%)	5 (22)	2 (5)	0.088
Knapp classification^c^			0.722
I (%)	3 (13)	4 (10)	
II (%)	0 (0)	2 (5)	
III (%)	20 (87)	35 (85)	
Age at the operation (range) (y)	11.6 ± 9.7 (2.0–41.1)	5.0 ± 4.1 (1.0–18.1)	0.005*
Postoperative follow-up (range) (mo)	25.7 ± 19.2 (6.8–75.7)	29.8 ± 20.8 (6.3–72.8)	0.433

y = years; SOP = superior oblique palsy; PD = prism diopters; D = diopters; arcsec = seconds of arc; mo = months

Data are mean ± standard deviation (range) or n (%).

Asterisk * indicates statistical significance.

^a^: Of patients who had an angle of head tilt more than 5° according to our criteria. If a patient had both clinical and photograph measurement data, the maximum value of them was accepted.

^b^: The fraction indicates the proportion of the number of patients who had preoperative measurable data against the patients who exhibited abnormal head posture.

^c^: Limited distribution was due to surgical indication of IO myectomy in SOP.

Mean preoperative degree of head tilt was not different between groups, 9.4 ± 8.6 (range, 0–30.2)° in the present group vs. 14.6 ± 11.8 (range, 0–45)° in the absent group (*P* = 0.067). In the patients with abnormal head tilt defined by our criteria, 12.8 ± 8.3 (range, 5–30.2)° in the present group (16 patients) vs. 17.8 ± 10.9 (range, 5–45)° in the absent group (33 patients) (*P* = 0.109). The direction of head tilt was mostly toward the opposite side of palsy except 1 patient in the present group. Mean postoperative follow-up period was 25.7 ± 19.2 (range, 6.8–75.7) months in the present group and 29.8 ± 20.8 (range, 6.3–72.8) months in the absent group (*P* = 0.433).

Preoperative ocular motility measurements including the magnitude of hypertropia in the primary position, hypertropia during contralateral and ipsilateral gaze, and contralateral and ipsilateral tilt positions were similar in both groups. No significant differences were noted in the frequencies of ipsilateral overelevation in adduction, ipsilateral underdepression in adduction, contralateral pseudo-overaction of SO, and SR overaction/contracture in both groups.

### Surgical Outcome

At 1 month after operation, 22 (96%) of 23 patients in the present group were successfully aligned and 1 (4%) of 23 patients had overcorrection. In the absent group, motor success at 1 month after operation was found in 39 (95%) of 41patients and 2 (5%) were undercorrected (*P* = 0.276). At postoperative 12 months, all patients had motor success in the present group. On the contrary, undercorrection of ipsilateral hypertropia was found in 6 (15%) patients only in the absent group andmotor success was achieved in 35 (85%) patients (*P* = 0.080). After a mean follow-up of 28.3 ± 20.2 months, 21 (91%) patients in the present group showed motor success at the last examination with no undercorrection or recurrence, and 2 (9%) patients resulted in overcorrection with masked bilateral SOP. In the absent group, motor success was achieved in 30 (73%) patients (*P* = 0.002), while 11 (27%) patients showed undercorrection of hypertropia. Of them, 2 patients showed initial undercorrection at the postoperative 1 month, while the rest of them were recurred after minimum 6 months of an initial motor success. The amount of ipsilateral hypertropia in the primary gaze was significantly greater in the absent group (2.7 ± 5.2 PD) compared with the present group (-0.2 ± 2.1 PD) at the last follow-up examination (*P* = 0.002). Final overcorrection occurred in 2 (9%) patients of the present group and both of them were revealed as masked bilateral SOP at postoperative 17 and 26 months, respectively. Except those 2 patients, all patients with a normal trochlear nerve obtained final motor success. Conversely, there was no overcorrection and masked bilateral SOP in the absent group until the last examination.

Head tilt improvement after an average follow-up of 28.3 ± 20.2 months postoperatively was found in 13 (81%) of 16 patients in the present group and 25 (68%) of 37 patients in the absent group (*P* = 0.508). Mean angle of head tilt after an average follow-up of 28.3 ± 20.2 months postoperatively was 0.4 ± 2.3° in the present group (17 patients) versus 3.2 ± 7.1° in the absent group (35 patients) (*P* = 0.039). In the patients showed the preoperative head tilt according to our criteria, the angle of head tilt was significantly greater in the absent group (3.3 ± 7.4°) compared to the present group (0.2 ± 2.6°) at the last follow-up visit (*P* = 0.042).

**Fig 1:**
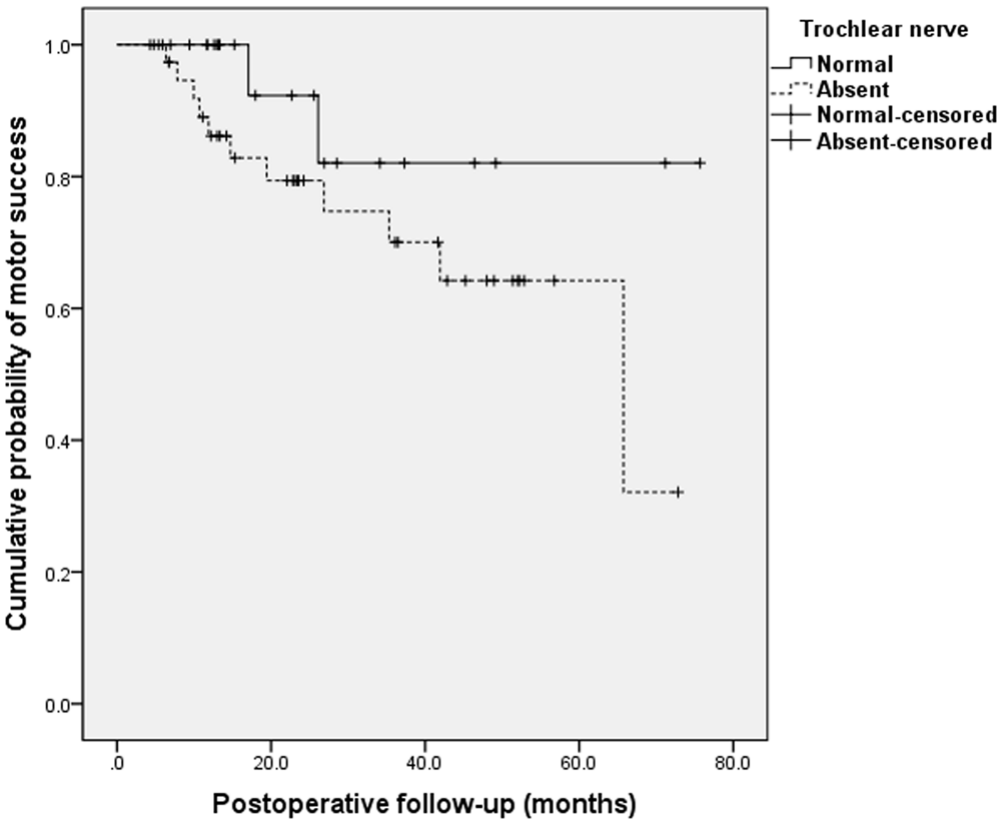
Kaplan-Meier survival plots of motor success after inferior oblique myectomy in superior oblique palsy. The cumulative probabilities of motor success at 24 months after surgery were 86% in patients without a trochlear nerve by Kaplan-Meier analysis. In patients with a trochlear nerve, cumulative probabilities of motor success at 24 months were 92%and all cases of failure were revealed as masked bilateral SOP. There was no significant difference in the cumulative probabilities of overall success at postoperative 24 months between both groups (*P* = 0.138, log-rank test).

**Fig 2:**
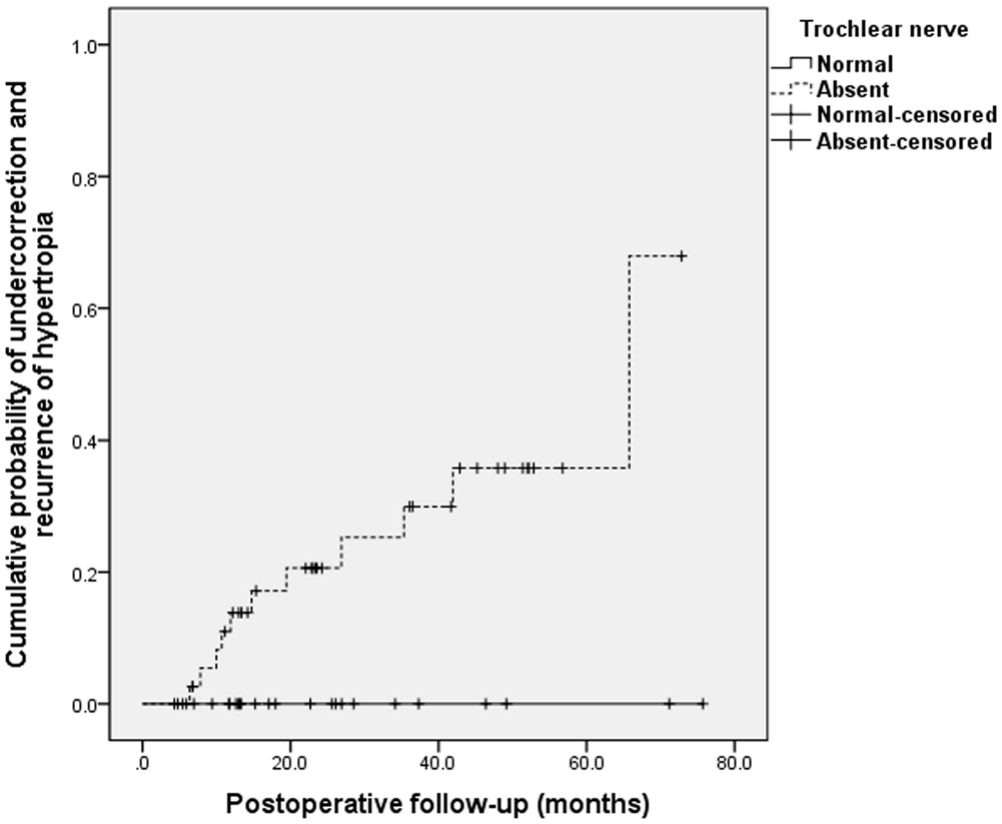
Kaplan-Meier survival plots of undercorrection of hypertropia after inferior oblique myectomy in superior oblique palsy. The cumulative probabilities of undercorrection and recurrence of hypertropia at postoperative 24 months were 21% in the absent group, contrary to 0% in the present group (*P* = 0.014, log-rank test).

**Fig 3:**
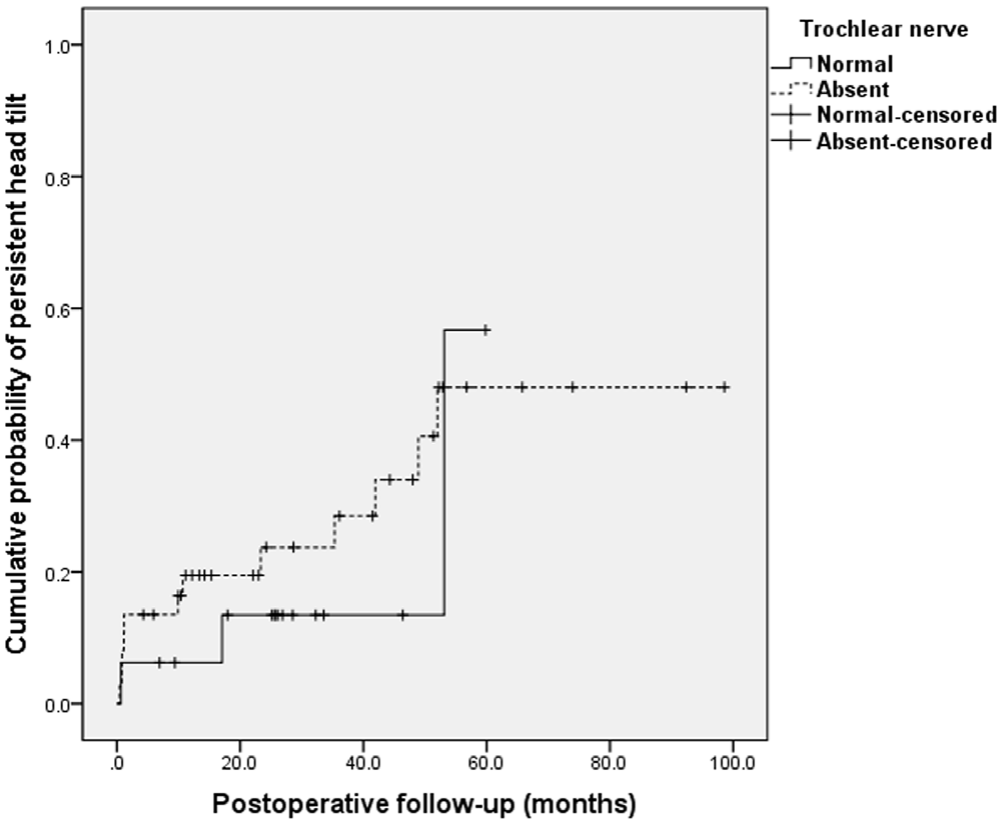
Kaplan-Meier survival plots of persistent head tilt after inferior oblique myectomy in superior oblique palsy. The cumulative probabilities of persistent head tilt at 24 months after surgery were 20% in patients without a trochlear nerve. The patients with a trochlear nerve showed 14% at 24 months after surgery. (*P* = 0.486, log-rank test).

The cumulative rates of motor success at 24 months after surgery were 86% in the absent group versus 92% in the present group (*P* = 0.138, log-rank test; Fig 1). However, after excluding the 2 patients of masked bilateral SOP (9%) as a confounding factor, pure motor success rates of the present group were 100% throughout the follow-up period (*P* = 0.018, log-rank test). Furthermore, the cumulative probabilities of undercorrection and recurrence of hypertropia at postoperative 24 months were 21% in the absent group against 0% in the present group (*P* = 0.014, log-rank test; Fig 2). The cumulative probabilities of persistent head tilt at 24 months after surgery were 20% and in patients without a trochlear nerve and 14% in patients with a trochlear nerve at 24 months after surgery. The cumulative probabilities of persistent head tilt increased over time in both groups by similar rates (*P* = 0.486, log-rank test; Fig 3).

Comparison of postoperative changes in ocular deviation and other ocular motility parameters at postoperative months 1, 12, and at the last follow-up examination were analyzed. The mean amount of change in vertical deviation and degree of head tilt corrected by IO myectomy were similar in the present and absent group. Other ocular motility parameters including ipsilateral overelevation in adduction, ipsilateral underdepression in adduction, contralateral overelevation in adduction and the amount of surgical correction of each parameter were similar in both groups throughout the observation period. Reoperation for undercorrection or recurrence was required in 9 (22%) of 41 patients in the absent group.

### Factors related to Surgical Outcomes

Table 2 noted the factors associated with the 2 main surgical outcomes; motor failure which is represented by undercorrection and recurrence of ipsilateral hypertropia, and persistent head tilt. Earlier age at operation was associated with reduced probabilities of motor success and head tilt improvement (*P* = 0.009, *P* = 0.022 respectively).

**Table 2:** Cox proportional hazards model in Motor Failure and Persistent Head Tilt.

Predictor	Motor failure^a^			Persistent head tilt		
	HR	Standard deviation	*P*-value	HR	Standard deviation	*P*-value
Group						
Present group						
Absent group	3.0	0.8	0.157	1.6	0.7	0.490
Age at the operation	0.6	0.2	0.009*	0.7	0.1	0.022*
Head tilt angle	1.1	0.0	0.074	1.1	0.0	0.038*

Cox proportional hazards model with firth's correction.

Asterisk * indicates statistical significance.

^a^: Motor failure was defined as the undercorrection and recurrence of ipsilateral hypertropia after the inferior oblique myectomy.

The amount of preoperative angle of head tilt was a significant risk factor related with persistent head tilt after operation. Greater preoperative angle of head tilt was associated with low probabilities of head tilt resolution after surgery (*P* = 0.038). The absence of the trochlear nerve was found to be insignificant factor influencing motor failure (*P* = 0.157) and persistent head tilt (*P* = 0.490).

## Discussion

This study is the first to compare the surgical outcomes of IO myectomy between congenital SOP with or without a trochlear nerve over a mean follow-up period of 2 years. In this study, congenital SOP without a trochlear nerve showed higher rates of undercorrection and recurrence after IO myectomy compared with those who had a trochlear nerve.

Several proposed causes for the failure of IO weakening surgery to address SO palsy include anatomic or structural abnormalities, insufficient surgery when part of the IO muscle is missed, and a very poor SO function. [25, 26] We precisely isolated the total IO muscle from adjacent anatomic structures during surgery and conducted the Guyton’s exaggerated traction test to confirm complete IO myectomy at the end of the operation. In the 9 reoperated patients with undercorrection or recurrence of ipsilateral hypertropia, only 1 patient had adherence near the operated site, otherwise the others showed persistent SO weakness on Guyton’s exaggerated traction test. Therefore, in the present study, the higher incidence of undercorrection and recurrence of ipsilateral hypertropia in the absent group during the postoperative period can be explained by the distinct etiology of the trochlear nerve. In this study, 64% of the patients with congenital SOP demonstrated absence of the trochlear nerve, and all these patients showed variable degrees of SO muscle hypoplasia, whereas the other patients had a normal trochlear nerve and SO muscle. As already presented in our former study, absence of the trochlear nerve can be verified as a congenital cranial dysinnervation disorder (CCDD) accompanied by denervation atrophy of the SO muscle. In contrast, patients with a trochlear nerve presented normal and symmetric muscle volume, therefore it can be assumed to have functional abnormalities such as abnormal SO muscle tendons or pulley dysfunctions. [2] These 2 distinguishable pathologic mechanisms, the neurologic denervation and the end organ abnormality might respond differently to the same operation. The suggestion can be supported by the significant disparities of cumulative probabilities of undercorrection and recurrence of hypertropia depending on the presence of the trochlear nerve, which were documented over the time-course by Kaplan-Meier survival analysis (*P* = 0.014, log-rank test). Even though the absence of the trochlear nerve was a weak risk factor of undercorrection and recurrence in Cox proportional hazards model with firth's correction (HR 3.0, *P* = 0.157), it can be postulated that the absence of the trochlear nerve might be associated with at least earlier undercorrection and recurrence of ipsilateral hyperdeviation. Additionally, the mean age at operation was a predictive factor for the undercorrection and recurrence of hypertropia (HR 0.6, *P* = 0.009) which was remarkably earlier in the absent group rather than present group (*P* = 0.005). Older age at the primary operation was correlated with increased probabilities of motor success. This might indicate that the earlier onset of decompensation which need prompt surgical intervention imply innate lower capacities of fusion for maintenance of good surgical outcome.

Regarding head tilt improvement, the mean angle of head tilt was significantly more reduced in the present group at the last examination (0.4 ± 2.3 (range, -5.1 to 7.4)° compared to the absent group (3.2 ± 7.1 (range, -5.8 to 29.0)°, *P* = 0.039) even though the overall rates of head tilt improvement were not different in both groups. According to the previous literature, the compensatory head tilt posture in unilateral SOP was associated with the effect of reducing the vertical deviation rather than to decrease ocular torsion. [27] It might be assumed that patients with an absent trochlear nerve had a potentially weaker SO tonus that results in a more compensatory effort to minimize their vertical deviation. This finding consists with the simultaneous increase of ipsilateral hypertropia and head tilt angle at the final examination in the absent group.

The success rates in both groups are not lower than the previous reports on the effect of IO myectomy to correct head tilt (53–90%), both in the present group (81%) and the absent group (68%). [9, 11, 15] Overall improvement of head tilt in this study is relatively higher than previous literatures involving young children. [28, 29] Some authors noticed about 50% of failure after single IO weakening surgery in congenital SOP infants with a mean age of 15.5 (range, 8–24) months. [28] However, the previous studies had performed IO myotomy or IO recession as a weakening procedure, so the higher success rates for correcting head tilt in this study might result from the different procedure, a complete IO myectomy. The absent group consists of children with a mean age of 2.7 ± 2.9 years, so we regard IO myectomy as an effective procedure to address head tilt correction in extremely young children with congenital SOP, and most of them had an absent trochlear nerve in this study. Predictable factor analysis for persistent head tilt were similar to motor failure; greater age at first operation was a positive predictor for head tilt improvement after surgery (HR = 0.7, *P* = 0.022). This result also supports our suggestion that earlier decompensation from neurologic imbalance has negative effects on surgical outcome. Additionally, a greater preoperative angle of head tilt suggests a weaker fusional vergence, which was also revealed as a risk factor for persistent head tilt (HR = 1.1, *P* = 0.038). Thus, it is apparent that better results can be obtained with proper surgical intervention especially in patients with an early onset or large amount of head tilt who may have inborn innervational defects such as absence of the trochlear nerve.

There are some considerable limitations in this study. Because this study was a retrospective one, the possibility for selection bias and confounding issues remain. An important selection bias is the exclusion of patients with SR muscle contracture. Patients with SR contracture or large hyperdeviation requiring multiple muscle surgeries were not included in our study. Most of the patients who had a tight SR underwent combined ipsilateral SR recession. Contracture of the ipsilateral SR muscle is frequently accompanied with unilateral SOP up to 46%, mostly when the clinical course is long-standing with a large magnitude of hyperdeviation, regardless of the absence of the trochlear nerve. [2, 21] These patients mostly underwent combined muscle surgery and the surgical outcome tended to be worse than those who were indicated for single IO myectomy. [30, 31] As severe manifesting patients were mostly excluded, the results of our study cannot be extrapolated to these patients. In our study, 78% (50/64) showed a maximum vertical deviation of ≤ 20 PD and 22% of patients with hypertropia of ≥ 25 PD (14/64) were selectively included if there was no evidence of SR contracture. In the comparative analysis between 2 surgical outcomes between patients with hypertropia of < 25 PD and ≥ 25 PD, the results showed insignificant differences in motor success (39 (78%) of 50 patients with hypertropia of < 25 PD versus 12 (86%) of 14 patients with hypertropia of ≥ 25 PD, *P* = 0.715 by Fisher’s exact test) as well as head tilt improvement in patients who had preoperative head tilt (29 (69%) of 42 patients with hypertropia of < 25 PD versus 9 (82%) of 11 patients with hypertropia of ≥ 25 PD, *P* = 0.482 by Fisher’s exact test) between 2 groups. Secondly, the SO muscle and tendon were not anatomically explored in most of the patients and we could not identify structural abnormalities of patients with a normal trochlear nerve whether they had a lax SO muscle tendon, pulley dysfunction, or other abnormalities. However, we conducted the intraoperative Guyton’s exaggerated traction test to detect prominent SO laxity. Finally, as formerly mentioned, the discrimination for the real “absence” of a trochlear nerve in MRI can be affected by technical limitation or motion artifacts. [2] Therefore, we applied strict criteria for selecting or interpreting MRI images to overcome these invincible problems.

In conclusion, patients without a trochlear nerve showed significantly higher rates of undercorrection and recurrence of ipsilateral hypertropia after IO myectomy than those with a trochlear nerve. In contrast, head tilt improvement was similar, regardless of the presence of the trochlear nerve. Younger age at first operation was correlated with both motor failure and persistent head tilt. Greater degree of preoperative compensatory head tilt was a risk factor for persistent head tilt after surgery.
